# Involvement of the protein kinase Akt2 in insulin-stimulated Rac1 activation leading to glucose uptake in mouse skeletal muscle

**DOI:** 10.1371/journal.pone.0212219

**Published:** 2019-02-08

**Authors:** Nobuyuki Takenaka, Natsumi Araki, Takaya Satoh

**Affiliations:** Laboratory of Cell Biology, Department of Biological Science, Graduate School of Science, Osaka Prefecture University, Sakai, Osaka, Japan; Universidad Pablo de Olavide, SPAIN

## Abstract

Translocation of the glucose transporter GLUT4 to the sarcolemma accounts for glucose uptake in skeletal muscle following insulin administration. The protein kinase Akt2 and the small GTPase Rac1 have been implicated as essential regulators of insulin-stimulated GLUT4 translocation. Several lines of evidence suggest that Rac1 is modulated downstream of Akt2, and indeed the guanine nucleotide exchange factor FLJ00068 has been identified as an activator of Rac1. On the other hand, the mechanisms whereby Akt2 and Rac1 are regulated in parallel downstream of phosphoinositide 3-kinase are also proposed. Herein, we aimed to provide additional evidence that support a critical role for Akt2 in insulin regulation of Rac1 in mouse skeletal muscle. Knockdown of Akt2 by RNA interference abolished Rac1 activation following intravenous administration of insulin or ectopic expression of a constitutively activated phosphoinositide 3-kinase mutant. The activation of another small GTPase RalA and GLUT4 translocation to the sarcolemma following insulin administration or ectopic expression of a constitutively activated form of phosphoinositide 3-kinase, but not Rac1, were also diminished by downregulation of Akt2 expression. Collectively, these results strongly support the notion that Rac1 acts downstream of Akt2 leading to the activation of RalA and GLUT4 translocation to the sarcolemma in skeletal muscle.

## Introduction

The glucose transporter GLUT4 is responsible for insulin-dependent glucose uptake in skeletal muscle and adipose tissue [1–3]. GLUT4 is stored in specific intracellular compartments termed GLUT4 storage vesicles in unstimulated cells, and vesicles containing GLUT4 molecules are transported toward the plasma membrane in response to insulin stimulation. Subsequently, GLUT4 is redistributed to the plasma membrane through fusion of GLUT4-containing vesicles with the plasma membrane, and permits blood glucose to be incorporated into the cell across the plasma membrane. Following insulin stimulation, various signaling pathways for the induction of the plasma membrane translocation of GLUT4 are activated downstream of the insulin receptor. A key component of this insulin signaling is a kinase cascade consisting of phosphoinositide 3-kinase (PI3K) and its downstream protein kinases, PDK1 and Akt2. Phosphorylation of various substrate proteins by activated Akt2 is thought to be a prerequisite for the induction of GLUT4 translocation.

Recent studies have shown that the Rho family small GTPase Rac1 plays an important role in insulin-dependent glucose uptake in skeletal muscle [4–11]. Involvement of Rac1 in insulin-dependent glucose uptake was originally reported in cultured myoblasts and myotubes [5–7, 10], and then confirmed in mouse skeletal muscle [9, 11]. Impaired glucose tolerance and higher plasma insulin concentrations after intraperitoneal glucose injection in muscle-specific rac1 knockout (m-rac1-KO) mice actually demonstrate the physiological importance of Rac1 in insulin action in skeletal muscle [9].

Although the mechanisms whereby Rac1 is activated following insulin stimulation have been extensively explored by the use of cultured myoblasts and mouse skeletal muscle, our understanding of the mechanisms remains incomplete. Rac1 was indeed activated after ectopic expression of a constitutively activated mutant of PI3K or Akt2 in L6 myoblasts and mouse gastrocnemius muscle fibers [12–14]. In addition, these constitutively activated mutants induced plasma membrane translocation of GLUT4 in wild-type, but not m-rac1-KO, mouse gastrocnemius muscle fibers [13]. Therefore, it is conceivable that Rac1 is regulated downstream of Akt2 in skeletal muscle insulin signaling.

The guanine nucleotide exchange factor (GEF) that regulates the GTP/GDP state of Rac1 downstream of the insulin receptor was also explored, and the Dbl family GEF FLJ00068 (also termed PLEKHG4 or puratrophin-1) was identified as such a regulatory molecule originally in L6 myoblasts [10, 13, 15]. The role of FLJ00068 in the activation of Rac1 downstream of the insulin receptor was further verified in mouse skeletal muscle. A constitutively activated mutant of FLJ00068 indeed stimulated GLUT4 translocation in skeletal muscle of wild-type, but not m-rac1-KO, mice [15]. Moreover, Rac1 activation and GLUT4 translocation caused by ectopic expression of a constitutively activated mutant of PI3K or Akt2 were completely abrogated by small interfering RNA (siRNA)-mediated knockdown of FLJ00068 in mouse skeletal muscle [16].

Collectively, we thought that the most likely mechanism for Rac1 activation in insulin signaling depends on the GEF FLJ00068, which may be regulated downstream of Akt2. In contrast, another model in which Rac1 is regulated downstream of PI3K, but not Akt2, and Akt2 and Rac1 act in parallel to each other for exocytosis of GLUT4-containing vesicles and cytoskeletal rearrangements, respectively, is also proposed [4, 17, 18]. Therefore, further evidence supporting the role for Akt2 upstream of Rac1 is required. Actually, we have not yet tested Rac1 activation and plasma membrane translocation of GLUT4 in Akt2-deficient mouse skeletal muscle due to unavailability of Akt2 knockout mice in our laboratory. However, we recently established siRNA-mediated knockdown and in situ detection of Rac1 activation in mouse skeletal muscle [14, 16, 19], which enabled us to directly examine the involvement of Akt2 in insulin-stimulated activation of Rac1. In this study, we aim to provide additional in vivo evidence for the involvement of Akt2 in Rac1 activation in skeletal muscle insulin signaling by using a mouse model.

## Materials and methods

### Materials

A rat monoclonal antibody against the hemagglutinin (HA) epitope tag (11 867 423 001), a mouse monoclonal antibody against the Myc epitope tag (05–724), and rabbit polyclonal antibody against the V5 epitope tag (V8137) were purchased from Roche Applied Science (Germany), Merck Millipore (MA, USA), and SIGMA-Aldrich (MO, USA), respectively. A goat polyclonal antibody against Akt2 (AF23151) was purchased from R&D systems (MN, USA). Mouse monoclonal antibodies against Rac1 (610650) and RalA (610221) were purchased from BD Biosciences (CA, USA). A mouse monoclonal antibody against α-tubulin (T9026) was purchased from SIGMA-Aldrich. Antibodies against goat IgG, mouse IgG, rabbit IgG, and rat IgG conjugated with CF 350/543/647 were purchased from Biotium (CA, USA). A sheep polyclonal antibody against mouse IgG conjugated with horseradish peroxidase (NA9310) was purchased from GE Healthcare (UK). Insulin was purchased from Eli Lilly (IN, USA). Two siRNA duplexes against mouse Akt2, #1 (Genosys (MO, USA), Mm_AKT2_4936; 5´-GAGAUGUGGUGUACCGUG-3´) and #2 (Genosys, Mm_AKT2_4937; 5´-GACUCUUCCACAUCUGAG-3´), and a mixture of non-targeting control (NC) siRNA duplexes (Dharmacon (CO, USA), D-001206-13; Duplex 1, 5´-AUGAACGUGAAUUGCUCAAUU-3´; Duplex 2, 5´-UAAGGCUAUGAAGAGAUACUU-3´; Duplex 3, 5´-AUGUAUUGGCCUGUAUUAGUU-3´; and Duplex 4, 5´-UAGCGACUAAACACAUCAAUU-3´) were commercially obtained.

### Animal experiments

Conventional 12-week-old male mice with the C57BL/6 genetic background were used in this study. This study was carried out in strict accordance with the recommendations in the Guidelines for Proper Conduct of Animal Experiments of the Science Council of Japan. The protocol was approved by the Committee on the Ethics of Animal Experiments of Osaka Prefecture University. Surgery was performed under anesthesia by intraperitoneal injection of a solution of medetomidine, midazolam, and butorphanol as described below, and all efforts were made to minimize suffering.

### Quantitative reverse transcription-polymerase chain reaction (RT-PCR) analysis

The total cellular RNA was isolated from mouse gastrocnemius muscle using the Sepasol-RNA I Super G (Nacalai (Japan)) according to the manufacturer’s instructions. cDNAs were synthesized using the SuperScript IV first-strand synthesis system for RT-PCR (Termo Fisher Scientific (MA, USA)). PCR was carried out using TB Green Premix Ex Taq II (Takara Bio (Kyoto, Japan)) and specific primers (Termo Fisher Scientific) (5´-TGTGATGGAGTATGCCAACG-3´ and 5´-CTCCAGAGCTGACACAATCT-3´ for Akt2, and 5´-ATGAAGATCAAGATCATTGCTCCTC-3´ and 5´-ACATCTGCTGGAAGGTGGACAG-3´ for β-actin) with Thermal Cycler Dice Real Time System III (Takara Bio) according to the manufacturer’s instructions. The mRNA levels were determined by the comparative Ct method followed by normalization with the β-actin mRNA level in each cDNA sample.

### Immunoblot analysis

Mouse gastrocnemius muscle proteins were separated by SDS-polyacrylamide gel electrophoresis and transferred on to a 0.45-μm pore size polyvinylidene difluoride membrane (GE Healthcare). Membranes were incubated with primary and horseradish peroxidase-conjugated secondary antibodies. Specific proteins were visualized by Chemi-Lumi One Ultra (Nacalai). Images were captured, and densitometric analysis was carried out by using a chemiluminescence imaging system (Ez-Capture MG, Atto (Japan)).

### Gene transfer into mouse gastrocnemius muscle by electroporation

Plasmid DNAs and siRNAs were introduced into mouse gastrocnemius muscle by electroporation as previously described [16]. Adult male C57BL/6 mice were anesthetized by intraperitoneal injection of a solution of medetomidine (0.3 mg/kg of body weight), midazolam (4.0 mg/kg of body weight), and butorphanol (5.0 mg/kg of body weight). A combination of expression plasmids (pCAGGS-GLUT4*myc*7-green fluorescent protein (GFP) [11], pCAGGS-a myristoylated form of the PI3K catalytic subunit p110α (Myr-p110α)-HA^×^3 [13], and pCAGGS-HA^×^3-Rac1(G12V) [11]) (80 μg in total) and siRNA duplexes (NC, #1, or #2) (1.7 μg in total) were dissolved in 50 μl of 9 mg/ml NaCl and injected longitudinally into gastrocnemius muscle with a 27-gauge needle. A pair of stainless steel electrode needles fixed 5 mm apart were then inserted into the muscle belly, and square wave electrical pulses (50 milliseconds) were applied three times (100 V, 90 V, and 81 V, respectively) at 100-millisecond intervals (for poring) using a pulse generator (NEPA21 Type II, Nepa Gene (Japan)). Subsequently, square wave electrical pulses (50 milliseconds) were applied three times (20 V, 12 V, and 7.2 V, respectively) at 100-millisecond intervals followed by three more pulses under the same conditions except that the polarity is opposite (for transfer).

### Isolation of mouse gastrocnemius muscle fibers and detection of activated forms of Rac1 and RalA

Mouse gastrocnemius muscle fibers were isolated, and activated forms of Rac1 and RalA were detected as previously described [16,19]. Mice were fasted for 16 h and anesthetized by intraperitoneal injection of a solution of medetomidine (0.3 mg/kg of body weight), midazolam (4.0 mg/kg of body weight), and butorphanol (5.0 mg/kg of body weight) 5 days after gene transfer by electroporation. Insulin was then administered intravenously. Gastrocnemius muscle was excised from anesthetized mice and fixed with 40 mg/ml paraformaldehyde in phosphate-buffered saline (PBS) for 40 min on ice. Individual muscle fibers were teased from fixed muscle with fine forceps under stereomicroscopy on ice and fixed in overlay assay buffer (50 mM Hepes-NaOH (pH 7.3), 150 mM NaCl, 20 mM MgCl2, and 0.05% (v/v) Tween 20) supplemented with 20 mg/ml paraformaldehyde on ice for 1 min. Thereafter, muscle fibers were treated with a glutathione S-transferase (GST) fusion of the V5 epitope-tagged Rac1-binding domain of POSH (GST-POSH(251–489)-V5^×^3) or a GST fusion of the V5 epitope-tagged RalA-binding domain of Sec5 (GST-V5^×^3-Sec5(1–99)) (10 μg/ml; purified from Escherichia coli transformants as described in Ref. 10) in overlay assay buffer supplemented with 0.1% (v/v) Triton X-100 and 50 μg/ml bovine serum albumin on ice for 20 min. After washing three times with overlay assay buffer, muscle fibers were fixed again in overlay assay buffer supplemented with 20 mg/ml paraformaldehyde on ice for 5 min. Fixed muscle fibers were washed with PBS supplemented with 0.05% (v/v) Tween 20 three times and incubated with an antibody against the V5 tag (for the detection of GST-POSH(251–489)-V5×3 or GST-V5^×^3-Sec5(1–99)). Muscle fibers were counterstained with antibodies against Rac1 or RalA, Akt2, and the HA tag (for the detection of Myr-p110α). Primary antibodies were subsequently detected with fluoresceinated secondary antibodies. Images were obtained and analyzed using a confocal laser-scanning microscope (FV1200, Olympus). Fluorescent intensities were quantified using ImageJ software. The activity of Rac1 or RalA was estimated by the ratio of V5 and Rac1 or RalA fluorescent intensities (V5/Rac1 or V5/RalA). Values of 6 muscle fibers in total from 3 different mice under each condition were used for statistical analysis (Student's t test).

### Detection of activated forms of Rac1 and RalA in frozen sections of mouse gastrocnemius muscle

Activated forms of Rac1 and RalA were detected in frozen sections of mouse gastrocnemius muscle as previously described [16]. Mice were fasted for 16 h and anesthetized by intraperitoneal injection of a solution of medetomidine (0.3 mg/kg of body weight), midazolam (4.0 mg/kg of body weight), and butorphanol (5.0 mg/kg of body weight) 5 days after gene transfer by electroporation. Insulin was then administered intravenously. Gastrocnemius muscle was excised from anesthetized mice, fixed in 40 mg/ml paraformaldehyde in PBS for 30 min on ice, and frozen in OCT compound (Sakura Finetek (USA)). All the frozen sections were made in approximately the same sections of the muscle. Frozen sections were fixed in overlay assay buffer supplemented with 20 mg/ml paraformaldehyde on ice for 1 min and treated with GST-POSH(251–489)-V5^×^3 or GST-V5^×^3-Sec5(1–99) (10 μg/ml) in overlay assay buffer supplemented with 0.1% (v/v) Triton X-100 and 50 μg/ml bovine serum albumin on ice for 40 min. After washing three times with overlay assay buffer, frozen sections were fixed again in overlay assay buffer supplemented with 20 mg/ml paraformaldehyde on ice for 5 min. Fixed frozen sections were washed with PBS supplemented with 0.05% (v/v) Tween 20 three times and incubated with an antibody against the V5 tag (for the detection of GST-POSH(251–489)-V5^×^3 or GST-V5^×^3-Sec5(1–99)). Frozen sections were counterstained with antibodies against Rac1 or RalA, Akt2, and the HA tag (for the detection of Myr-p110α). Primary antibodies were subsequently detected with fluoresceinated secondary antibodies. Images were obtained and analyzed using a confocal laser-scanning microscope (FV1200, Olympus). Fluorescent intensities were quantified using ImageJ software. The activity of Rac1 or RalA was estimated by the ratio of V5 and Rac1 or RalA fluorescent intensities (V5/Rac1 or V5/RalA). Values of 6 frozen sections in total from 3 different mice under each condition were used for statistical analysis (Student's t test).

### Isolation of mouse gastrocnemius muscle fibers and GLUT4 reporter assay

Mouse gastrocnemius muscle fibers were isolated, and GLUT4 reporter assay was performed as previously described [11,13,15,16,19]. The GLUT4 reporter GLUT4*myc*7-GFP was originally described in Ref. 20. Mice were fasted for 16 h and anesthetized by intraperitoneal injection of a solution of medetomidine (0.3 mg/kg of body weight), midazolam (4.0 mg/kg of body weight), and butorphanol (5.0 mg/kg of body weight) 5 days after gene transfer by electroporation. Insulin was then administered intravenously. Gastrocnemius muscle was excised from anesthetized mice and fixed with 40 mg/ml paraformaldehyde in PBS for 40 min. Individual muscle fibers were teased from fixed muscle with fine forceps under stereomicroscopy and incubated in 10 mg/ml bovine serum albumin in PBS for more than 30 min. Thereafter, muscle fibers were treated with an anti-Myc tag antibody (for the detection of GLUT4*myc*7-GFP translocated to the sarcolemma) for 1 h and washed three times with PBS. Subsequently, muscle fibers were permeabilized with 0.1% (v/v) Triton X-100 in PBS for 10 min, washed three times with 0.1% (v/v) Tween 20 in PBS, and incubated in 0.1% (v/v) Tween 20 in PBS supplemented with Mouse Ig Blocking Reagent (Vector Laboratories (CA, USA)) for 1 h. Muscle fibers were then treated with antibodies against Akt2 and the HA tag (for the detection of Myr-p110α and Rac1(G12V)) overnight at 4°C and washed three times with PBS. Primary antibodies were subsequently detected with fluoresceinated secondary antibodies. Images were obtained and analyzed using a confocal laser-scanning microscope (FV1200, Olympus). Fluorescent intensities were quantified using ImageJ software. The relative amount of GLUT4*myc*7-GFP translocated to the sarcolemma was estimated by the ratio of Myc and GFP fluorescent intensities (Myc/GFP). Values of 6 muscle fibers in total from 3 different mice under each condition were used for statistical analysis (Student's t test).

## Results

### siRNA-mediated knockdown of Akt2 in mouse skeletal muscle

We recently reported siRNA-mediated efficient knockdown of signaling molecules, such as FLJ00068 [16] and RalA [19], in mouse skeletal muscle, which enabled us to assess whether these molecules are involved in insulin-dependent signal transduction even if genetically modified mice are unavailable. In this study, we took advantage of this significant methodological improvement to obtain evidence supporting a critical role of Akt2 in insulin-dependent activation of Rac1 and subsequent signaling events.

**Fig 1:**
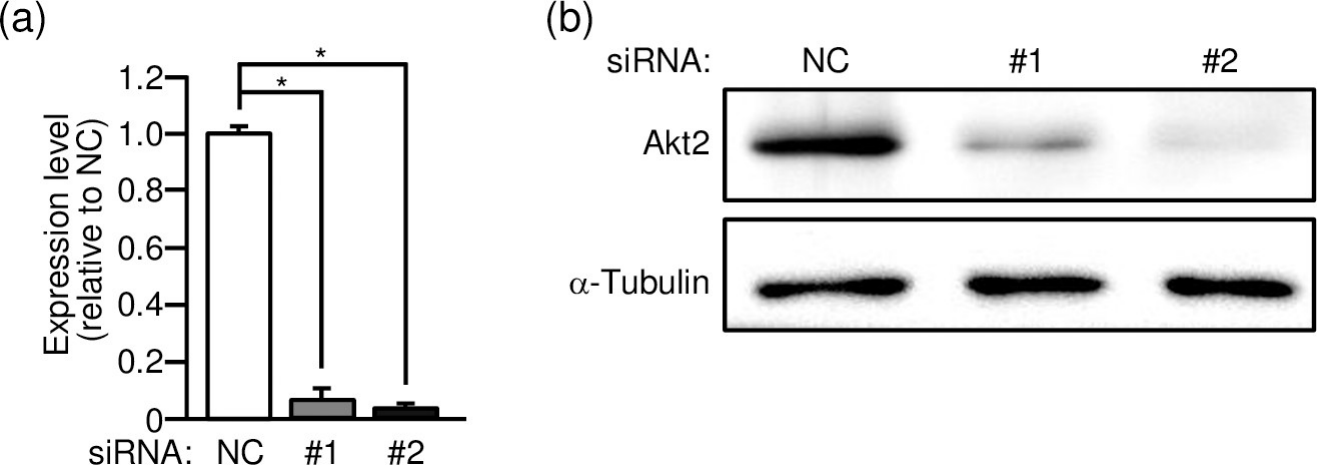
Knockdown of Akt2 by RNA interference in gastrocnemius muscle. (a) Two siRNA duplexes against mouse Akt2 (#1 and #2) and a mixture of NC siRNA duplexes were introduced into gastrocnemius muscle fibers of wild-type mice. The expression level of the akt2 mRNA was assessed by quantitative RT-PCR analysis. The expression levels relative to that in NC siRNA-treated muscle are shown as means ± S.E. (n = 6). *P < 0.001. (b) The expression levels of Akt2 and α-tubulin proteins were assessed by immunoblot analysis.

Two different siRNA duplexes against Akt2 (#1 and #2) were individually introduced into gastrocnemius muscle of wild-type mice, and the expression level of the akt2 mRNA was assessed by quantitative RT-PCR analysis (Fig 1A). The akt2 mRNA level was lowered to approximately 10% of the control level after treatment with either of the specific siRNA duplexes. Correspondingly, the Akt2 protein level as determined by immunoblot analysis was significantly lowered in Akt2 siRNA-treated skeletal muscle fibers (Fig 1B).

### Effect of Akt2 knockdown on the activation of Rac1

**Fig 2:**
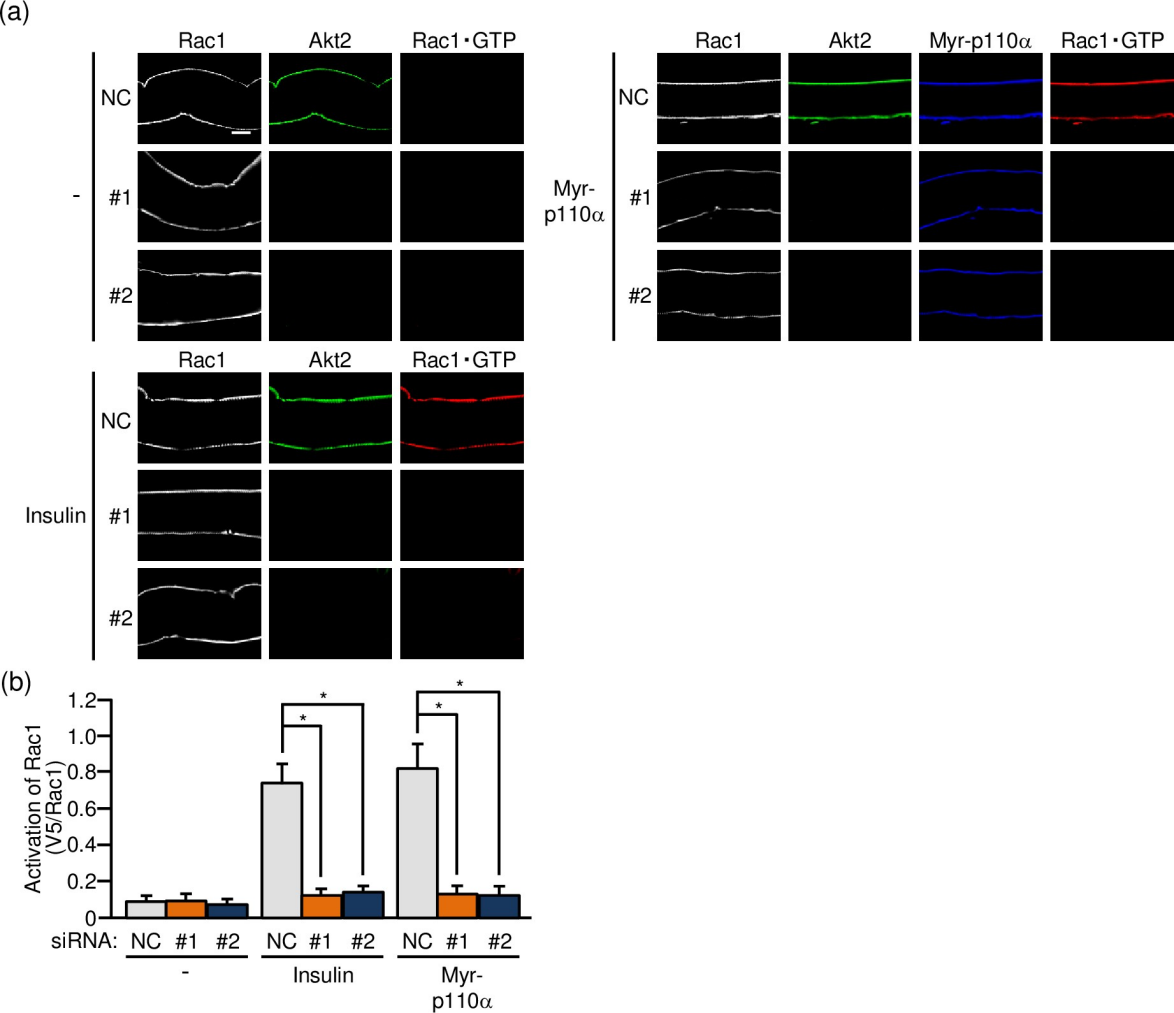
Akt2-dependent activation of Rac1 in mouse gastrocnemius muscle as detected by immunofluorescent microscopy of isolated gastrocnemius muscle fibers. (a) The expression vector for Myr-p110α, together with either one of two siRNA duplexes against mouse Akt2 (#1 and #2) and a mixture of NC siRNA duplexes, was introduced into gastrocnemius muscle of wild-type mice. Insulin (175.5 μg/kg body weight) was administered intravenously. Endogenous Rac1 and Akt2 were visualized by immunofluorescent staining with anti-Rac1 and anti-Akt2 antibodies, respectively. Myr-p110α was visualized by immunofluorescent staining with an anti-HA antibody. Activated Rac1 (Rac1·GTP) was visualized by immunofluorescent staining with an anti-V5 antibody after treatment with GST-POSH(251–489)-V5^×^3. Scale bar, 20 μm. (b) Activation of Rac1 shown in (a) was quantified. Gray, orange, and blue bars represent the treatment with NC, #1, and #2 siRNA duplexes, respectively. Data are shown as means ± S.E. (n = 6). *P < 0.001.

As a step to confirm that Akt2 is in fact implicated in insulin-dependent Rac1 activation, the effect of Akt2 knockdown on the activation state of Rac1 was examined in gastrocnemius muscle. Firstly, the activated form of Rac1 (Rac1·GTP) in mouse gastrocnemius muscle was detected by immunofluorescent microscopy using a polypeptide probe, which specifically recognizes activated Rac1 and is detected by fluoresceinated secondary antibodies, in isolated paraformaldehyde-fixed muscle fibers [14–16]. The activation of Rac1 following intravenous injection of insulin was not detected after siRNA-mediated knockdown of Akt2 whereas Rac1 was significantly activated in control muscle fibers (Fig 2). PI3K is a key signaling component downstream of the insulin receptor, and ectopic expression of a constitutively activated mutant of PI3K (Myr-p110α) induced Rac1 activation (Fig 2). Rac1 activation in response to ectopic expression of constitutively activated PI3K was also highly sensitive to the inhibitory effect of Akt2 knockdown (Fig 2). These results provide evidence that Akt2 is involved in the regulation of Rac1 downstream of PI3K in insulin signaling.

**Fig 3:**
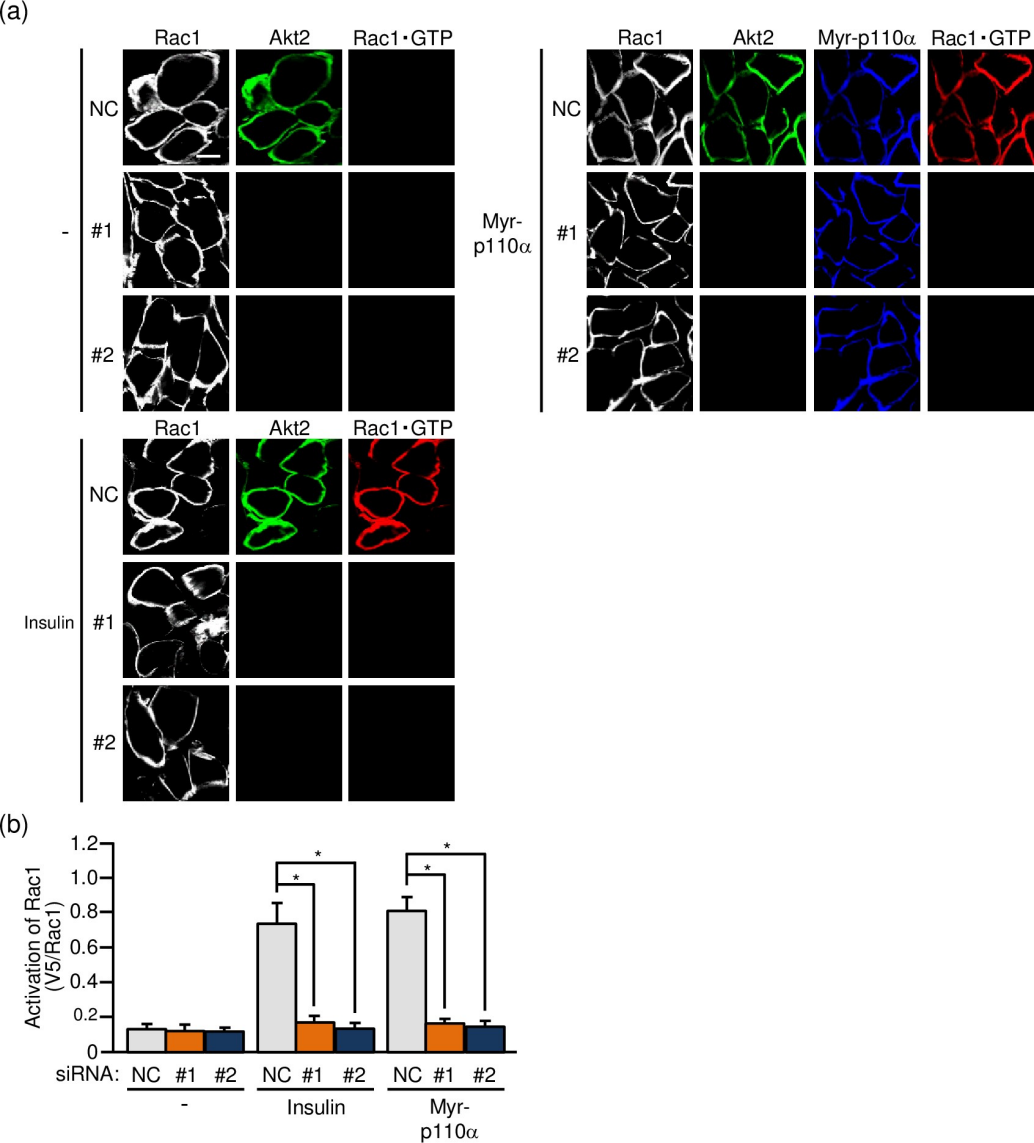
Akt2-dependent activation of Rac1 in mouse gastrocnemius muscle as detected by immunofluorescent microscopy of cross sections of gastrocnemius muscle. (a) The expression vector for Myr-p110α, together with either one of two siRNA duplexes against mouse Akt2 (#1 and #2) and a mixture of NC siRNA duplexes, was introduced into gastrocnemius muscle of wild-type mice. Insulin (175.5 μg/kg body weight) was administered intravenously. Endogenous Rac1 and Akt2 were visualized by immunofluorescent staining with anti-Rac1 and anti-Akt2 antibodies, respectively. Myr-p110α was visualized by immunofluorescent staining with an anti-HA antibody. Activated Rac1 (Rac1·GTP) was visualized by immunofluorescent staining with an anti-V5 antibody after treatment with GST-POSH(251–489)-V5^×^3. Scale bar, 20 μm. (b) Activation of Rac1 shown in (a) was quantified. Gray, orange, and blue bars represent the treatment with NC, #1, and #2 siRNA duplexes, respectively. Data are shown as means ± S.E. (n = 6). *P < 0.001.

Secondly, the effect of Akt2 knockdown on the activation of Rac1 was assessed in frozen sections of gastrocnemius muscle. We recently reported immunofluorescent microscopy using the above described activation-specific Rac1 probe in frozen sections of gastrocnemius muscle [16], and this method was also employed in the present study. Actually, insulin-stimulated Rac1 activation as observed in control muscle sections was totally diminished in Akt2 siRNA-treated muscle sections (Fig 3). Likewise, constitutively activated PI3K-induced Rac1 activation was also inhibited (Fig 3). These results further support the notion that Akt2 plays an important role in the regulation of Rac1 downstream of PI3K in skeletal muscle insulin signaling.

### Effect of Akt2 knockdown on the activation of RalA

**Fig 4:**
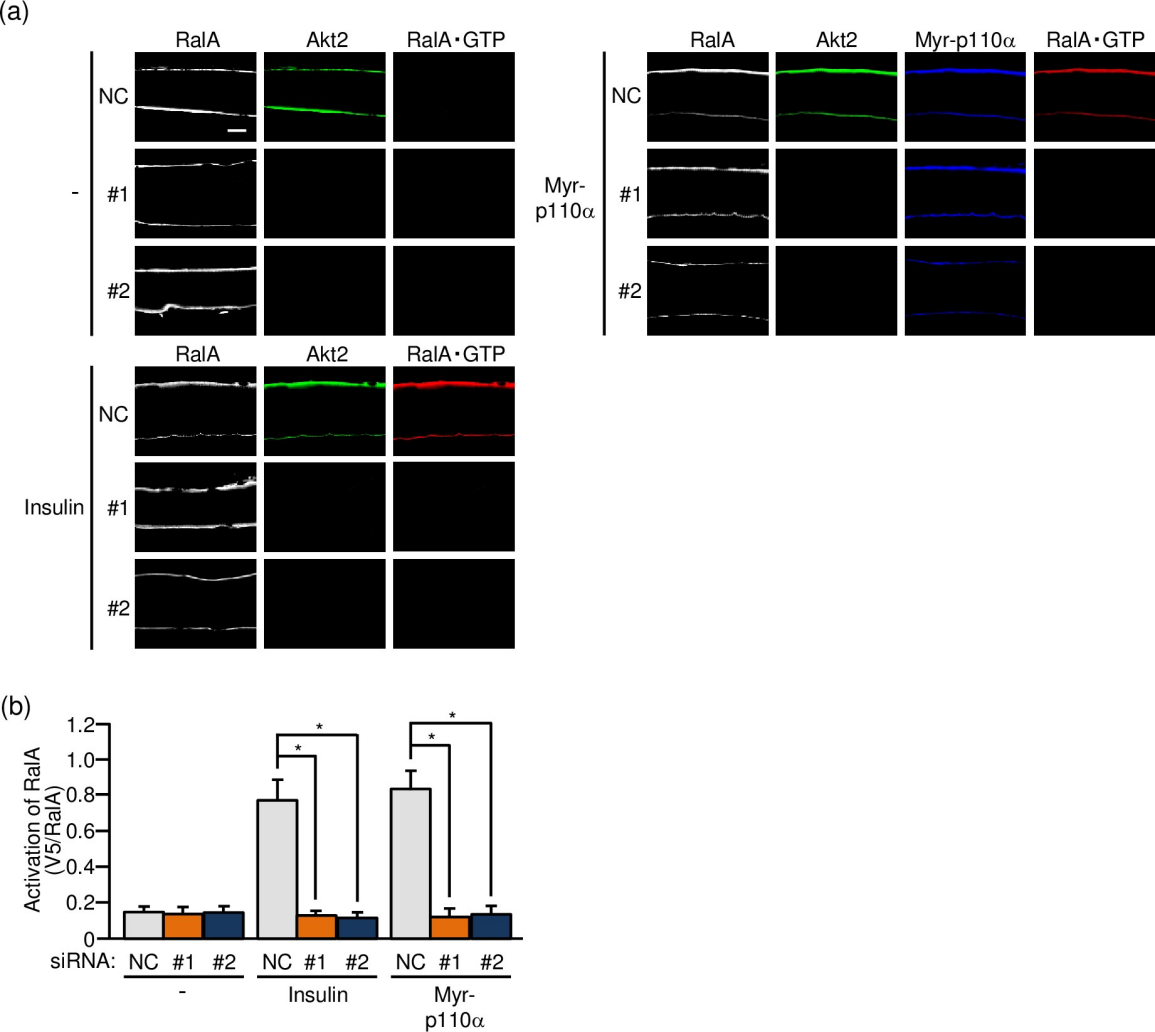
Akt2-dependent activation of RalA in mouse gastrocnemius muscle as detected by immunofluorescent microscopy of isolated gastrocnemius muscle fibers. (a) The expression vector for Myr-p110α, together with either one of two siRNA duplexes against mouse Akt2 (#1 and #2) and a mixture of NC siRNA duplexes, was introduced into gastrocnemius muscle of wild-type mice. Insulin (175.5 μg/kg body weight) was administered intravenously. Endogenous RalA and Akt2 were visualized by immunofluorescent staining with anti-RalA and anti-Akt2 antibodies, respectively. Myr-p110α was visualized by immunofluorescent staining with an anti-HA antibody. Activated RalA (RalA·GTP) was visualized by immunofluorescent staining with an anti-V5 antibody after treatment with GST-V5^×^3-Sec5(1–99). Scale bar, 20 μm. (b) Activation of RalA shown in (a) was quantified. Gray, orange, and blue bars represent the treatment with NC, #1, and #2 siRNA duplexes, respectively. Data are shown as means ± S.E. (n = 6). *P < 0.001.

The GTPase RalA belongs to the Ras family and has been implicated as a regulator of GLUT4 translocation in adipocytes and skeletal muscle [19–21]. In skeletal muscle, it has been demonstrated that RalA acts downstream of Rac1 [19,21], and therefore insulin-dependent RalA activation may also be suppressed when Akt2 is knocked down if Rac1 is really regulated downstream of Akt2. To test this hypothesis, we next visualized the activated form of RalA in paraformaldehyde-fixed gastrocnemius muscle fibers by immunofluorescent microscopy using an activation-specific polypeptide probe for RalA [16,19]. As expected, the activation of RalA following intravenous injection of insulin was substantially reduced when the expression of Akt2 was downregulated (Fig 4). Similarly, RalA activation following ectopic expression of constitutively activated PI3K was not detected in Akt2-knocked down muscle fibers (Fig 4).

**Fig 5:**
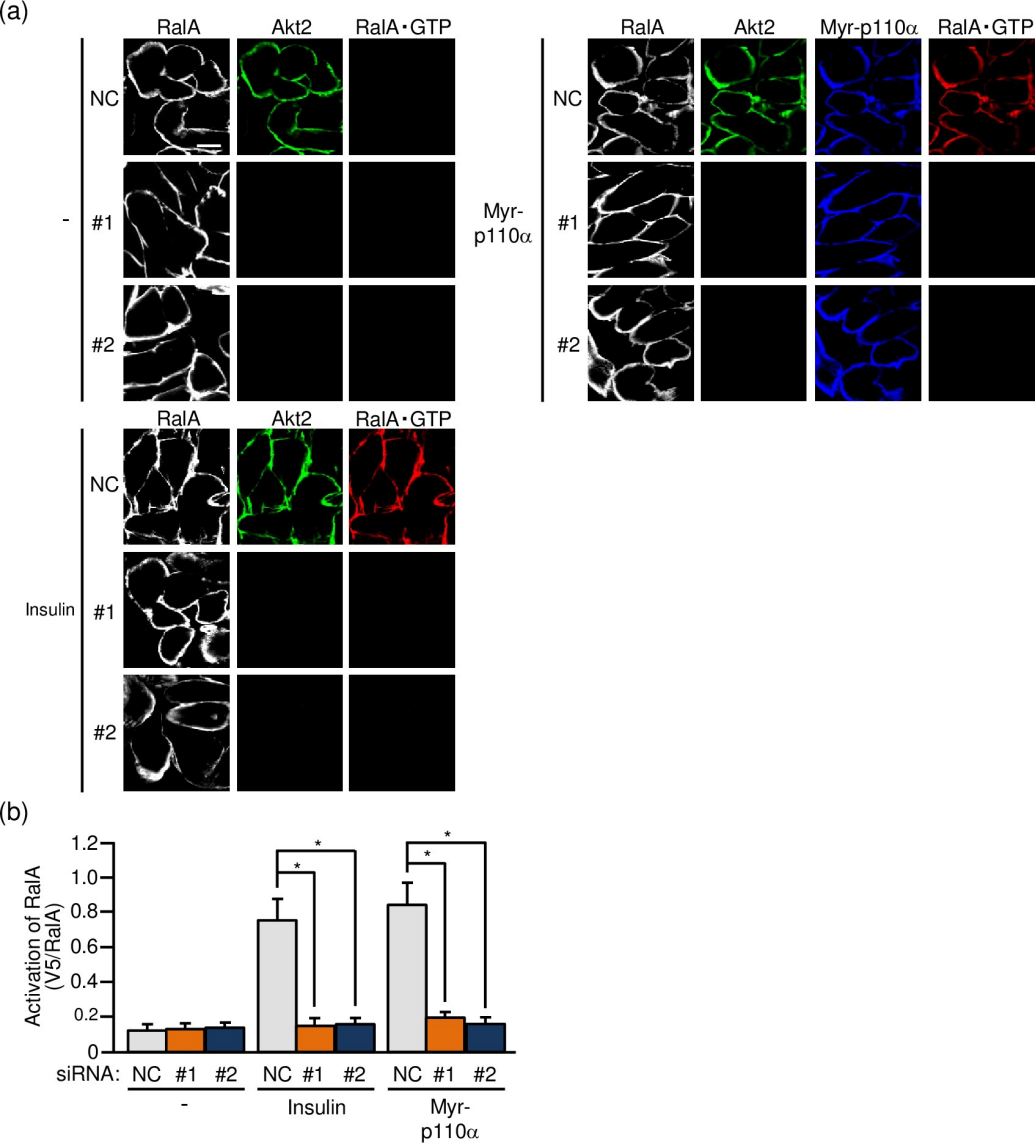
Akt2-dependent activation of RalA in mouse gastrocnemius muscle as detected by immunofluorescent microscopy of cross sections of gastrocnemius muscle. (a) The expression vector for Myr-p110α, together with either one of two siRNA duplexes against mouse Akt2 (#1 and #2) and a mixture of NC siRNA duplexes, was introduced into gastrocnemius muscle of wild-type mice. Insulin (175.5 μg/kg body weight) was administered intravenously. Endogenous RalA and Akt2 were visualized by immunofluorescent staining with anti-RalA and anti-Akt2 antibodies, respectively. Myr-p110α was visualized by immunofluorescent staining with an anti-HA antibody. Activated RalA (RalA·GTP) was visualized by immunofluorescent staining with an anti-V5 antibody after treatment with GST-V5^×^3-Sec5(1–99). Scale bar, 20 μm. (b) Activation of RalA shown in (a) was quantified. Gray, orange, and blue bars represent the treatment with NC, #1, and #2 siRNA duplexes, respectively. Data are shown as means ± S.E. (n = 6). *P < 0.001.

As in the case of Rac1, the effect of Akt2 knockdown on the activation of RalA was also assessed in frozen sections of gastrocnemius muscle. RalA activation in response to insulin stimulation or ectopic expression of constitutively activated PI3K was totally suppressed after siRNA-mediated knockdown of Akt2 (Fig 5). Taken together, these results provide evidence that RalA is regulated downstream of Akt2 in skeletal muscle insulin signaling.

### Effect of Akt2 knockdown on GLUT4 translocation to the sarcolemma

Finally, the effect of Akt2 knockdown on GLUT4 translocation to the sarcolemma was examined by the use of a GLUT4 reporter containing GFP and exofacial Myc tags (GLUT4*myc*7-GFP) [11,13,15,16,19,22]. The GLUT4 reporter was ectopically expressed in mouse skeletal muscle fibers via gene transfer, and insulin was administered intravenously. In some experiments, a constitutively activated form of PI3K (Myr-p110α) or Rac1 (Rac1(G12V)) was co-expressed with the GLUT4 reporter instead of insulin administration. The expression level of the GLUT4 reporter was estimated by green fluorescence signal intensity. Cell surface-translocated GLUT4 reporter molecules, which represent GLUT4 that is responsible for glucose transport, can be detected by immunofluorescent staining of the exofacial Myc tag.

**Fig 6:**
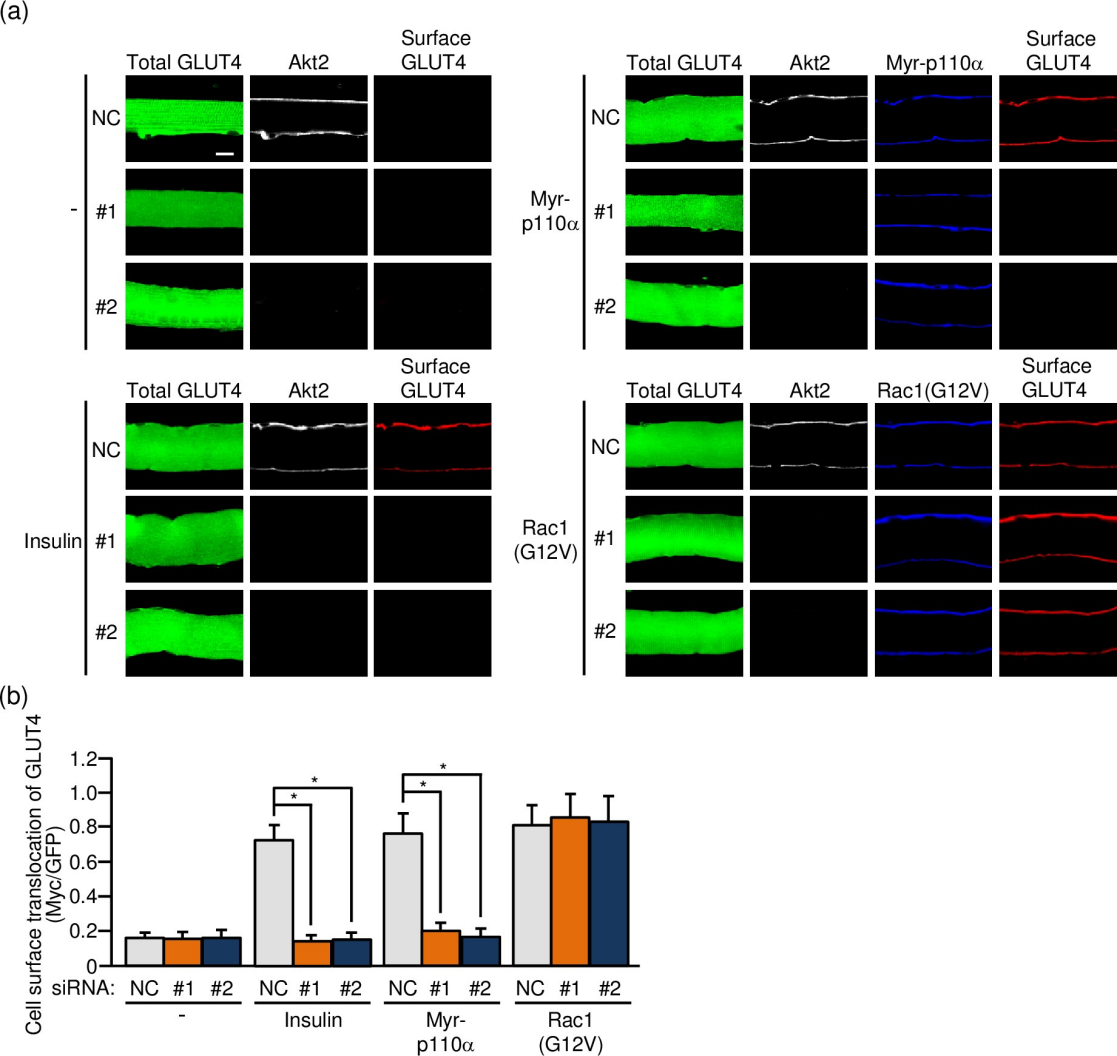
Inhibition of GLUT4 translocation to the sarcolemma by knockdown of Akt2. (a) Expression vectors for GLUT4*myc*7-GFP, Myr-p110α, Rac1(G12V), together with either one of two siRNA duplexes against mouse Akt2 (#1 and #2) and a mixture of NC siRNA duplexes, were introduced into gastrocnemius muscle of wild-type mice. Insulin (175.5 μg/kg body weight) was administered intravenously. Endogenous Akt2 was visualized by immunofluorescent staining with an anti-Akt2 antibody. Myr-p110α and Rac1(G12V) were visualized by immunofluorescent staining with an anti-HA antibody. GLUT4*myc*7-GFP translocated to the sarcolemma was visualized by immunofluorescent staining with an anti-Myc antibody before cell permeabilization. Scale bar, 20 μm. (b) Translocation of GLUT4*myc*7-GFP to the sarcolemma shown in (a) was quantified. Gray, orange, and blue bars represent the treatment with NC, #1, and #2 siRNA duplexes, respectively. Data are shown as means ± S.E. (n = 6). *P < 0.001.

Akt2 knockdown, in fact, significantly diminished insulin-dependent GLUT4 translocation to the sarcolemma (Fig 6). Furthermore, GLUT4 translocation induced by ectopic expression of constitutively activated PI3K was largely suppressed by Akt2 knockdown (Fig 6). In contrast, constitutively activated Rac1-induced GLUT4 translocation remained unchanged after Akt2 knockdown (Fig 6). Taken together, these results support the notion that Akt2 plays a critical role in insulin-dependent GLUT4 translocation to the sarcolemma, acting between PI3K and Rac1 in the signaling cascade.

## Discussion

Akt2 knockout impaired glucose uptake in isolated soleus and extensor digitorum longus muscles on exposure to a low concentration of insulin, and Akt2 knockout mice indeed exhibited hyperglycemia and glucose intolerance [23,24]. Therefore, it is widely accepted that Akt2 plays a pivotal role in the induction of GLUT4 translocation in insulin-stimulated skeletal muscle. Rac1 is also required for this process as described above [4–11]. However, detailed mechanisms for their regulation, particularly, the role of Akt2 in the activation of Rac1 remain incompletely understood.

Previously we demonstrated that a constitutively activated Akt2 mutant, when ectopically expressed, induced Rac1 activation [14] and GLUT4 translocation in a Rac1-dependent manner [13]. Although these results support the notion that Rac1 serves as a regulator of GLUT4 translocation downstream of Akt2, the effect of Akt2 knockout on putative downstream events, including Rac1 activation, remained to be examined because Akt2 knockout mice were not available in our laboratory. Recently, we succeeded in investigating the effect of siRNA-mediated downregulation of a particular signaling component, such as the GEF FLJ00068 [16] or the GTP-binding protein RalA [19] in gastrocnemius muscle fibers of living mice. We therefore sought to test whether knockdown of Akt2 affects insulin-dependent Rac1 activation and GLUT4 translocation. Moreover, the effect of Akt2 knockdown on the activation of RalA, which has been implicated as an essential signaling component downstream of Rac1, was examined. As described in this study, Akt2 is indeed a prerequisite for these signaling events, providing direct evidence that Akt2 is implicated in the regulation of Rac1, leading to the induction of GLUT4 translocation in skeletal muscle insulin signaling. These results are highly concordant with our previous data obtained by treatment with an Akt-specific inhibitor or siRNA-based knockdown of Akt2 in an L6 myoblast-derived cell line [12,13].

The GEF FLJ00068 has been implicated as a direct regulator of Rac1 downstream of Akt2 in mouse skeletal muscle based on the observation that Rac1 activation by constitutively activated Akt2 was abrogated when FLJ00068 was knocked down by siRNA treatment [16]. A critical role for this GEF in Akt2-dependent activation of Rac1 has also been demonstrated in an L6 myoblast-derived cell line [10, 15]. Furthermore, insulin-dependent subcellular redistribution of FLJ00068 to the plasma membrane was reported in both L6-derived cells and mouse gastrocnemius muscle [16]. This may be required for the activation of FLJ00068 because recruitment to the plasma membrane is a general mechanism for the activation of several types of Dbl family GEFs [25–28]. However, it remains largely unknown how Akt2 induces subcellular translocation and activation of FLJ00068. Following the activation of Akt2, currently unidentified post-translational modifications of FLJ00068 may occur, and subsequent protein-protein interactions of FLJ00068 with specific partners may direct subcellular translocation. Protein-protein interactions and subcellular translocation may disrupt an intramolecular inhibitory interaction, and thereby GEF activity may be increased. This model well explains the observation that deletion of the N-terminal portion conferred constitutive GEF activity on FLJ00068 [10]. Given that no consensus sequence motif for Akt substrates exists in FLJ00068, it seems unlikely that Akt2 directly phosphorylates FLJ00068, and rather unknown Akt2 substrates may interact with FLJ00068 or may induce modifications of FLJ00068. In future studies detailed mechanisms for Rac1 activation via FLJ00068 by Akt2 will be clarified.

Skeletal muscle is composed of heterologous muscle fibers with different metabolic characteristics, and the relative proportion of fiber types varies between muscles [29]. Type IIB fast twitch fibers, which contain a low level of oxidative enzymes, were reported to predominate in mouse gastrocnemius muscle [30]. In addition to type IIB fibers, type IIA, type IID, and hybrid fibers with high oxidative capacity exist in mouse gastrocnemius muscle [30]. In this study, we did not discriminate individual fiber types, and therefore, insulin response might vary even under the same assay conditions depending on muscle fiber types. Nevertheless, the results obtained showed reasonably small variations in insulin response, suggesting that insulin signaling in different muscle fiber types may be similar. In support of this notion, insulin stimulation of glucose uptake in type IIB, type IIA, type IID, and hybrid fibers was within the range of 5- to 10-fold [31]. Detailed examination of insulin signaling in different fiber types may provide further insights into the regulation of glucose uptake in response to insulin in skeletal muscle.

Insulin stimulation of glucose uptake, for which GLUT4 translocation from intracellular storage sites to the plasma membrane is responsible, occurs also in adipose tissue. The signaling mechanisms whereby insulin induces GLUT4 translocation are believed to be conserved, at least in part, between skeletal muscle and adipose tissue [1,8]. Therefore, Rac1 may lie downstream of Akt2, serving as a switch of insulin signaling that regulates GLUT4 translocation also in adipocytes. Data presented in a previous study by Marcusohn *et al*. argue against the involvement of Rac1 downstream of PI3K in insulin signaling of 3T3-L1 adipocytes [32]. In contrast, our recent study by the use of primary cultured mouse adipocytes provide evidence that insulin actually induces Rac1 activation in a PI3K-denpendent manner [33]. Future studies using genetically engineered adipocytes lacking Rac1 are needed to determine whether Rac1 acts downstream of Akt2 in insulin signaling for the induction of glucose uptake not only in skeletal muscle, but also in adipose tissue.

## Abbreviations

GEF: guanine nucleotide exchange factor
GFP: green fluorescent protein
GST: glutathione *S*-transferase
HA: hemagglutinin
m-*rac1*-KO: muscle-specific *rac1* knockout
Myr-p110α: a myristoylated form of the phosphoinositide 3-kinase catalytic subunit p110α
NC: non-targeting control
PBS: phosphate-buffered saline
PI3K: phosphoinositide 3-kinase
RT-PCR: reverse transcription-polymerase chain reaction
siRNA: small interfering RNA
